# Patient Position in Operative Endoscopy

**DOI:** 10.3390/jcm12216822

**Published:** 2023-10-28

**Authors:** Lino Polese, Emilia Giugliano, Michele Valmasoni

**Affiliations:** First Surgical Unit, Department of Surgery, Oncology and Gastroenterology, University of Padova, 35128 Padova, Italy; giugliano.emilia@gmail.com (E.G.); michele.valmasoni@unipd.it (M.V.)

**Keywords:** operative endoscopy, supine, prone, patient position, ESD, POEM, ERCP, colonoscopy

## Abstract

It is well known by surgeons that patient positioning is fundamental to exposing the organs when performing an operation via laparoscopy, as gravity can help move the organs and facilitate the exposure of the surgical site. But is it also important for endoscopic procedures? This paper examines various types of endoscopic operations and addresses the issue of the patient’s position. The patient’s position can be changed not only by rotating the patient along the head–toe axis but also by tilting the surgical bed, as is undertaken during laparoscopic surgical procedures. In particular, it is useful to take into account the effect of gravity on lesion exposure, tumour traction during dissection, crushing by body weight, risk of sample drop, risk of damage to adjacent organs, and anatomical exposure for procedures with radiological support. The endoscopist should always keep in mind the patient’s anatomy and the position of the endoscope during operative procedures, not limited to considering only intraluminal vision.

## 1. Introduction

It is well known by surgeons that patient positioning is fundamental to exposing the organs when performing an operation via laparoscopy, as gravity can help to move the organs and facilitate the exposure of the surgical site. But is it also important for endoscopic procedures? With the evolving role of operative endoscopy, it is fundamental to consider the aspect of the patient’s position. This paper examines various types of endoscopic operations and addresses the issue of patient positioning. 

## 2. Methods

This research was conducted by searching Medline for studies published until August 2023. The keywords used in the literature search included digestive endoscopy, colonoscopy, endoscopic retrograde pancreatography (ERCP), endoscopic submucosal dissection (ESD), peroral endoscopic myotomy (POEM), endosleeve, patient position, decubitus, left lateral, right lateral, prone, and supine.

The search was restricted to studies of human subjects published in peer-reviewed journals. The titles and abstracts of studies identified in the primary search were reviewed, and studies that did not address the research question were excluded. The full text of the remaining articles was reviewed to determine whether they contained relevant information. 

## 3. Results

**Figure 1:**
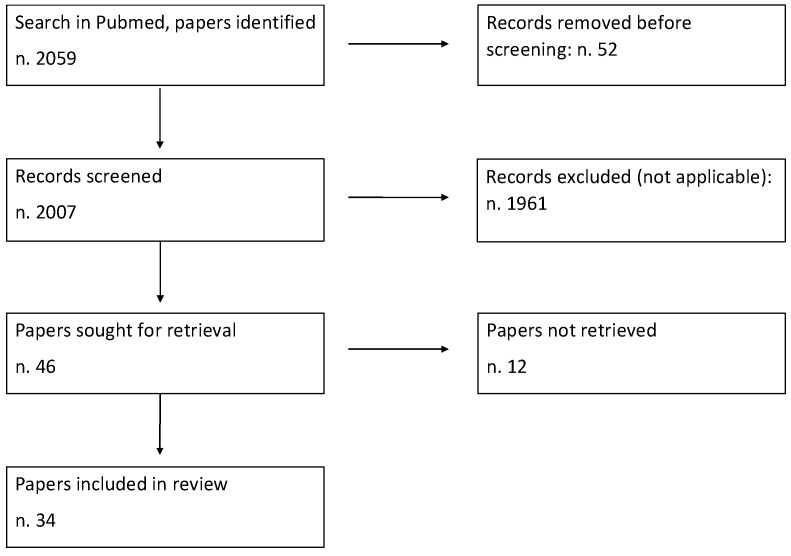
Study search and selection diagram.

The process of study selection for the review is presented in Figure 1.

## 4. Patient Position in Operative Endoscopy: General Aspects

### 4.1. Patient Position in Gastrointestinal Endoscopy

The standard positions adopted in gastrointestinal endoscopy are supine, prone, and left lateral. Right lateral is less commonly used but is reserved for particular cases, mainly in difficult colonoscopies. 

### 4.2. Possible Further Changes in Position Using Operating Beds

Most operating beds have the function of modifying the axis on which the patient rests while maintaining their stability. By modifying the angle of the head-to-foot axis, the Trendelenburg and anti-Trendelenburg positions are obtained.

**Figure 2:**
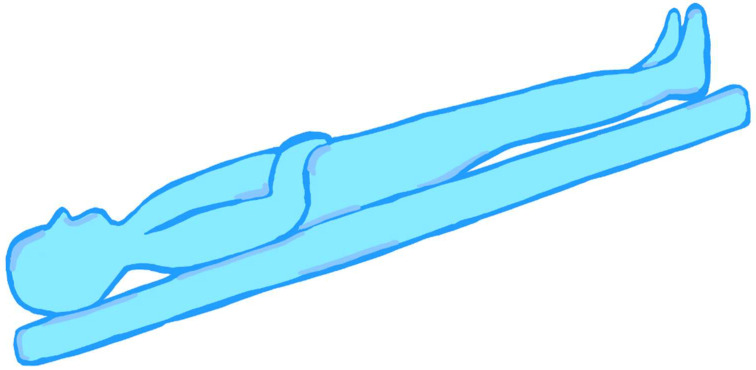
Trendelenburg.

Trendelenburg (Figure 2)

In the Trendelenburg position, the patient is supine on the table with their feet elevated above their head. This anti-shock position improves venous return in patients with low blood pressure, but it is also commonly used during laparoscopic surgery to move the small intestine away from the pelvis.

**Figure 3:**
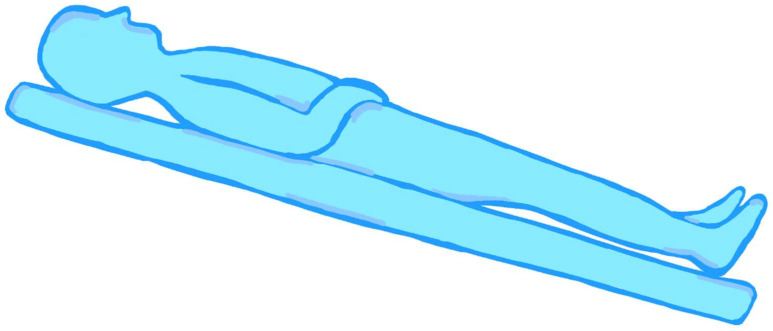
Anti-Trendelenburg position.

The anti-Trendelenburg or reverse-Trendelenburg position (Figure 3) is the opposite position and is used to move the bowel away from the upper abdominal region.

Thanks to the fixing systems attached to the patient’s bed, it is also possible to rotate the table to the right or left in order to obtain the most convenient position.

**Figure 4:**
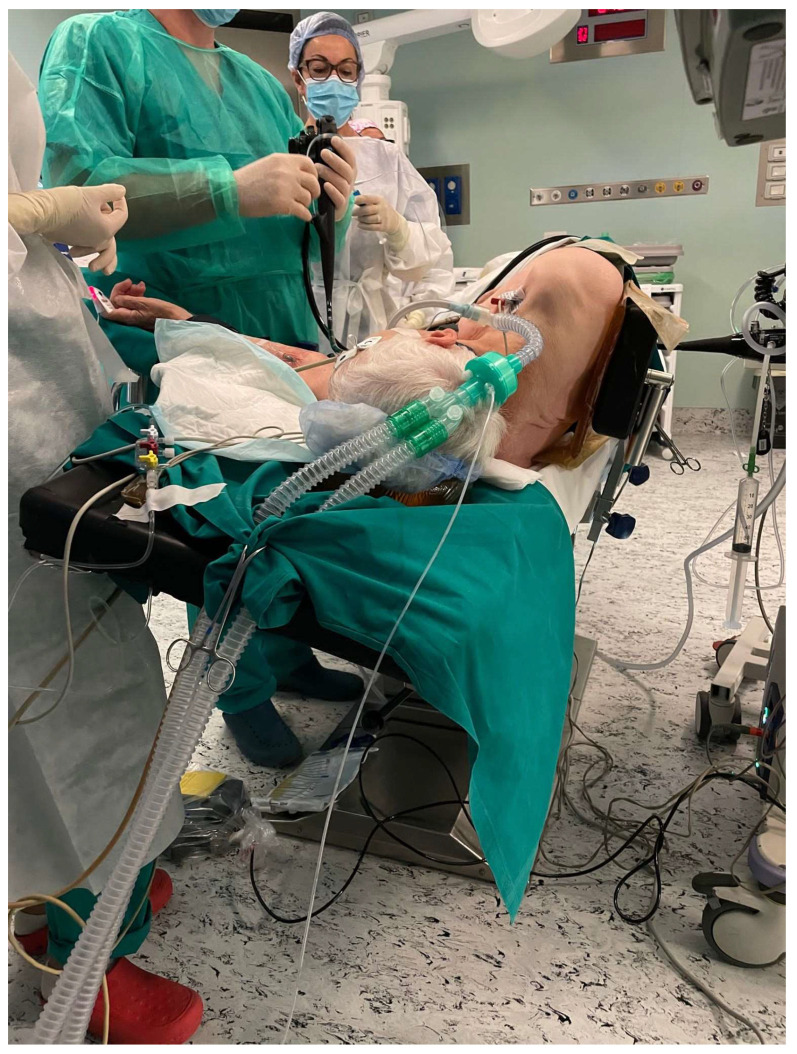
Left lateral position plus 15° angle rotation.

An example is presented in Figure 4. This picture shows the patient’s position during ESD for an extended antral gastric tumour. The tumour was located between the great curve and the posterior wall. The left lateral position with an additional 15° angle to the right facilitated the positioning of the tip of the endoscope over the lesion by gravity.

During laparoscopic surgery, the bed is rotated or lifted up to facilitate organ exposure through the effect of gravity. Gravity can also be used during operative endoscopy to facilitate endoscope positioning, help with lesion exposure, facilitate tumour traction during dissection, avoid crushing by body weight, mitigate the risk of sample drop or risk of damage to adjacent organs, and improve anatomical exposure for procedures with radiological support.

The endoscopist should always keep in mind the patient’s anatomy and the position of the endoscope during operative procedures, not limited to considering only intraluminal vision. 

### 4.3. Patient Limitations

Before positioning the patient, endoscopists must consider the possible limitations of the patient that might require a change in decubitus. According to Park et al., limitations for patient positioning include limited cervical movement, cervical cord injury, cervical spine operation, spine trauma, recent shoulder or hip surgery, pregnancy, and recent abdominal surgery [1].

### 4.4. Anaesthesiological Problems Related to the Patient’s Position

From an anaesthesiological point of view, the patient’s position could affect their respiratory function. Moreover, the prone position can make it more difficult and time-consuming to intubate the patient if this is deemed necessary.

As an example, ERCP is commonly performed in the prone position, which can make the placement of an emergency-advanced airway challenging. If conditions warrant an emergency endotracheal intubation during ERCP, this requires the immediate abortion of the procedure, the removal of patient restraints, the repositioning of fluoroscopy equipment, and, finally, the repositioning of the patient to supine, often from the fluoroscopy table onto a transport cart. This is a coordinated effort that may result in a delay when providing an advanced airway if not performed swiftly [2].

Although prone positioning can have positive effects on respiratory function (e.g., increases in functional residual capacity and the arterial partial pressure of oxygen) [3], considerations must be made to manage a patient needing an advanced airway during the procedure. 

Endoscopic submucosal dissection (ESD) under general anaesthesia in the left lateral position may lead to the transient impairment of a pulmonary function. For patients in the left lateral position undergoing ESD, the left lung is characterised by decreased ventilation and greater inhomogeneity. Performing the ESD procedure with carbon dioxide insufflation does not lead to significant changes in either regional ventilation or homogeneity. However, the changes in lung inhomogeneity during the ESD procedure are transient [4]. Taken together, these findings suggest temporary changes in lung status during the whole ESD. The main pathogenic mechanism is the development of atelectasis in the left lung areas and overdistension in the right lung areas [5].

During propofol sedation for colonoscopy, the left lateral position has shown a lower incidence of hypoxemia with respect to the supine position. In a randomised study by Klare P et al. [6], a lower incidence of hypoxemia was seen in the left lateral position with respect to supine during colonoscopy. Patients in the left lateral group showed lower apnoea rates (9.4% vs. 16.2%; *p* = 0.040) but had more episodes of hypotension (12.3% vs. 2.9%; *p* < 0.001). The incidence of hypoxemia was lower for the left lateral group than for the supine group in per-protocol analysis (1.8% vs. 11.2%; *p* = 0.003).

### 4.5. Fluid Pooling 

Fluid pooling can alter the visualisation of a lesion or source of bleeding [7].

In the case of a gastric fundal lesion, puddle formation can make it difficult to see a lesion when the patient is in the left lateral position. In such a case, changing the position from the left to right lateral allows for the better visualisation of the lesion with the assistance of gravity [8].

According to Tan Y et al. [9], a supine position during POEM makes the muscle incision easier for posterior myotomy because it does not require tip angulation, but the view can be limited due to fluid pooling.

**Figure 5:**
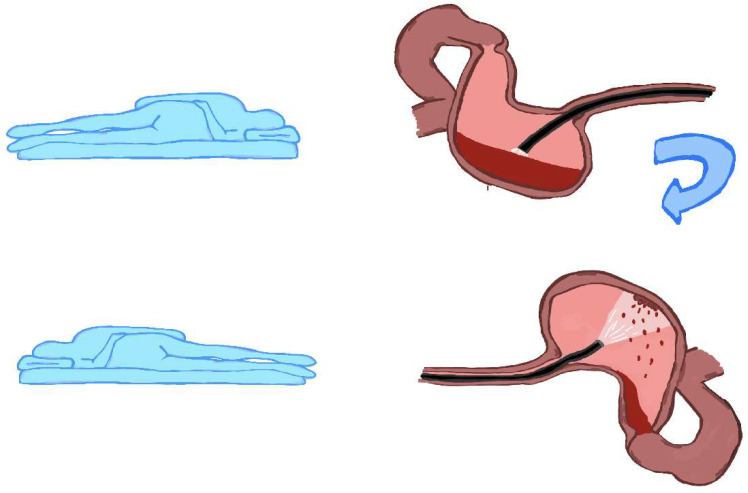
Changing the patient’s position can help expose the source of bleeding. In the first position, the blood covers the lesion, preventing its endoscopic visualisation. By rotating the patient from the left lateral position to the right lateral position, the blood moves, and the lesion becomes visible.

In an emergency, endoscopy is performed for bleeding from the upper digestive tract, and changing the patient’s position on the bed can be fundamental to exposing the source of the bleeding when there is blood collection over the lesion (Figure 5).

In the case of a patient intubated in a surgical bed, further modifications of the axis (Trendelenburg, anti-Trendelenburg, or right or left rotation) can further improve the visibility of the bleeding source.

In the “underwater” endoscopic submucosal dissection technique, on the contrary, the patient’s posture is changed to place the lesion at the lower side with regard to gravity, allowing the saline solution to accumulate around the lesion [10,11].

### 4.6. Organ Distension and Compression by Surrounding Organs

Digestive endoscopy consists of exploring hollow organs. The wall of the intestinal viscera can be compressed by the surrounding organs, and this can vary with the patient’s decubitus position. As an example, modifying the patient’s position during colonoscopy has been proposed as a simple and inexpensive technique to increase luminal distention [12].

In obese patients undergoing endoscopic sleeve gastroplasty, sporadic cases of gallbladder perforation by gastric stitches have been reported [13]. According to the authors, a risk factor is represented by the patient’s position during the procedure. In fact, it was reported that the patient was in the “swimmers” position instead of a more conventional supine, lazy left-lateral position, which may have brought the stomach and gallbladder into closer proximity, thus increasing the risk of gallbladder perforation with the use of a full-thickness gastric suturing technique. 

According to the authors, the suturing was erroneously started in close proximity to the lesser curvature of the stomach, which could have also increased the risk of biliary injury.

### 4.7. Endoscope Positioning by Gravity 

**Figure 6:**
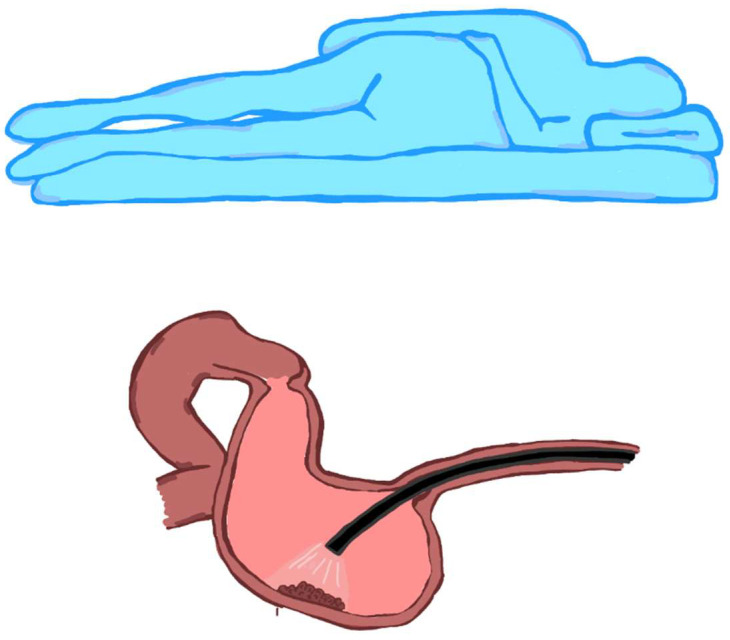
Endoscope positioning in the left lateral decubitus position: the tip of the endoscope falls to the greater gastric curvature. This leads the endoscope to naturally position itself on the lesion.

**Figure 7:**
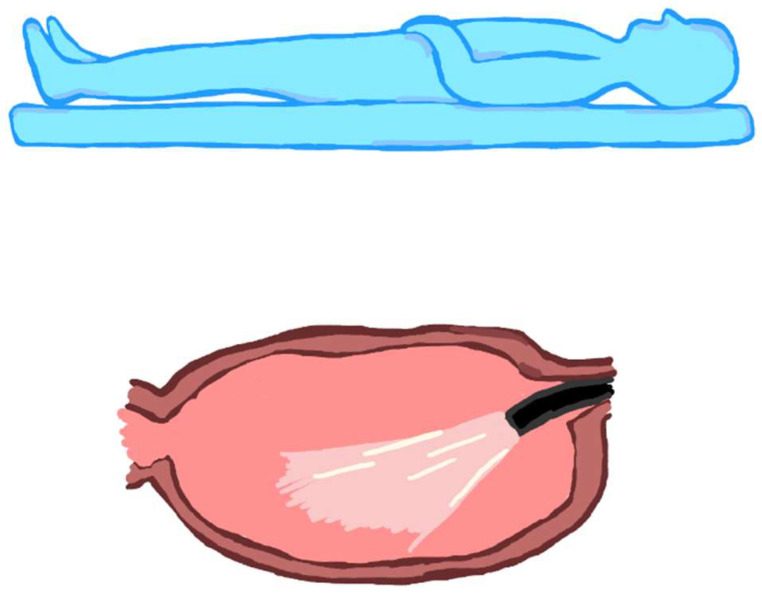
Endoscope positioning in the supine decubitus position: the tip of the endoscope falls to the posterior gastric wall.

More relevant for stomach lesions but worthy of consideration for other organs, including the oesophagus or the rectum, is the fact that the tip of the endoscope tends to position itself—by force of gravity—near the wall located on the side where the patient lies. Thus, if the patient is in the left lateral position, the endoscope’s tip will be positioned toward the greater stomach curvature (Figure 6); if the patient lies in the supine position, the endoscope tip will be positioned toward the posterior gastric wall (Figure 7). Clearly, fluid collects in the same position in the case of bleeding.

### 4.8. Effect of Gravity on Sampling

Another element subject to the force of gravity is sampling, both when the sample is partially detached during the ESD procedure and when it is completely removed. In the “underwater” ESD technique, the patient’s posture is changed to place the lesion at the lower side with respect to gravity to allow for saline solution accumulation around the lesion. This technique exploits the floating of the lesion to raise it with respect to the depth planes [10,11].

The effect of gravity on the sample must be considered in the case of duodenal lesion removal. In fact, if the patient is in a left lateral position, there is a greater risk that the sample will fall further downstream, with the risk of being lost. This happens less easily if the supine position is used instead.

### 4.9. Changing the Patient Position during a Procedure

The patient can be advantageously mobilised during an endoscopic procedure.

According to the aforementioned phenomena, it may be useful to start a procedure with the lesion at the lowest point of the lumen, but it may subsequently be useful to rotate the patient to mobilise a collected sample or mobilise the lesion using the force of gravity.

From this point of view, patient sedation is fundamental. A non-sedated or lightly sedated patient is easy to move and can cooperate with the process. Deeply sedated or anesthetised patients must be physically manipulated, increasing the procedure time and requiring significant coordination with endoscopy assistants. In a study by Ou et al., it took, on average, 44 s to complete position changes per examination during withdrawal in colonoscopy [14].

Nevertheless, polypectomy, after optimizing the patient’s position, is likely to be a much quicker process, more than compensating for the added time required to perform the move, as well as enhancing control and safety [15].

### 4.10. Endoscopist Position and Monitor Position

The position of the endoscopist is also crucial. The rotation of the endoscope, including the direction of the tip and the image, depends on the position of the endoscopist. For example, if the endoscopist moves from the patient’s left side to the patient’s right side, with the patient supine, an upside-down image is obtained.

The position of the monitor is also decisive because the endoscopist is forced to turn in the direction of the monitor, and this can influence the rotation plane of the endoscopic image. The availability of multiple monitors connected to a column facilitates a comfortable position for the endoscopist during various procedures.

### 4.11. Patient Position and Time Required for Procedures

Procedures on patients in the prone position can be more time-consuming with respect to those in the left lateral position.

According to a study by Issa D et al. [16], there is a reduction in room time for the supine with left lateral position with respect to the prone position. On average, there was a 38 min difference between supine and prone cases concerning the room turnover time. Therefore, supine patient positioning may increase endoscopy unit case volumes, accelerate patient access, and increase the departmental Relative Value Unit (RVU) reimbursement.

## 5. Patient Position for Specific Procedures: Upper-GI

### 5.1. POEM

Myotomy positions include the anterior (11–1 o’clock), posterior (5–7 o’clock), lateral greater curve (8 o’clock), and lateral lesser curve (3 o’clock). Anterior or posterior myotomy is more commonly used [17,18,19].

Currently, no evidence supports preferentially placing the patient in either the left lateral or supine position for the POEM procedure [20]. For some authors, the supine position is preferred anyway during posterior myotomy because the shaft tends to lie in a neutral position with less tension on the mucosal opening, particularly with advanced sigmoid achalasia. This prevents the inadvertent extension of the mucosal entry site resulting from extensive tension by the scope shaft. Moreover, in advanced sigmoid achalasia, in the left lateral position, the severity of angulations is greater because of the lateral exertion of gravity. This can increase the technical difficulty of submucosal tunnelling and subsequent myotomy. 

One limitation of working with the patient in the supine position for the posterior myotomy is the potential for fluid pooling posteriorly, which can obscure the working field or inhibit the application of spray coagulation. 

If an anterior myotomy is performed, the degree of the needle tip fling tends to be greater with the patient in the supine position. As a result, the length of each individual cut during the myotomy tends to be smaller to minimise the fling and prevent contralateral mucosal damage. This also likely has an impact on the procedure time [21]. 

### 5.2. PER ORAL ZENKER DIVERTICULOTOMY

Several endoscopic procedures can be performed for the treatment of Zenker diverticulum. These include rigid endoscopy and flexible endoscopy. The most recent of the flexible procedures is the ZPOEM, which borrows the submucosal tunnel technique of the POEM. Whether using the rigid or flexible diverticuloscope, the patient is usually positioned in the supine position [22]. This facilitates the exposure of the diverticulum and the operative area. 

### 5.3. ENDOSLEEVE

Endoscopic sleeve gastroplasty can be performed in both lateral and supine positions. 

As previously mentioned, in obese patients undergoing endoscopic sleeve gastroplasty, sporadic cases of gallbladder perforation by gastric stitches have been reported. According to the authors, a risk factor is represented by the patient’s position during the procedure [13]. In fact, it was reported that the patient was in the “swimmers” position instead of a more conventional supine, lazy, left-lateral position, which may have brought the stomach and gallbladder into closer proximity, thus increasing the risk of gallbladder perforation with the use of a full-thickness gastric suturing technique. According to the authors, suturing was erroneously started in close proximity to the lesser curvature of the stomach, which could have also increased the risk of biliary injury.

On the contrary, the left lateral position facilitates the positioning of the tip of the endoscope at the site of the operation: the great gastric curve. In any case, it must be considered that positioning an obese patient in the left lateral position and stabilizing them for an operation under general anaesthesia is challenging for the operating room staff and adds time to the procedure.

### 5.4. ERCP

In the initial period after its introduction, ERCP was performed with the patient in a left lateral position, but over time, the prone position has become the preferred position for most endoscopists performing this procedure. In fact, the prone position has been reported to improve the visualisation and cannulation of the ampulla of Vater [23]. The prone position also permits improved radiographic imaging of the biliary anatomy, reducing the risk of pancreatic duct cannulations [24]. 

In fact, fluoroscopic images of the bifurcation, right and left hepatic ducts, intrahepatic bile ducts, and pancreatic duct (PD) are adversely affected by the left lateral position [1,25]. According to a study by Issa D et al. [16], the success of incannulation was similar between the supine and prone positions. In their study, the authors prospectively evaluated patients undergoing ERCP performed by a supervised advanced endoscopy trainee (AET) at a tertiary care centre. Adult patients with native papillae were included. The AET was universally given five attempts per cannulation. Successful cannulation was achieved in 44 (69%) supine patients and in 17 (68%) prone patients (*p* = 0.95). Although the mean time to papilla cannulation was shorter in supine patients, the time to biliary cannulation (7.8 vs. 9.4 min; *p* = 0.53) and the number of attempts were similar.

According to a study by Park et al. [1], the left lateral position for ERCP is as effective and safe as the prone position. However, they reported a higher rate of unintended pancreatic duct (PD) cannulation and PD contrast injection; thus, they suggest that the left lateral position should be initially preferred for patients with limitations that increase the difficulty of prone positioning. Difficulties for prone positioning include cervical movement limitations due to cervical cord injury, cervical spine operations, or neck surgery; Parkinson’s disease; muscle contraction due to cerebral infarction; abdominal distension; ascites; recent abdominal surgery; severe obesity; and pregnancy.

Another particular situation that requires performing the procedure in the supine position is during collaboration with radiologists or surgeons. The simultaneous execution of the ERCP with radiologic percutaneous transhepatic biliary drainage or laparoscopic cholecystectomy requires the patient to be in the supine position.

Finally, as previously discussed, the prone position can make the placement of an emergency advanced airway challenging. In fact, if conditions warrant emergency endotracheal intubation during ERCP, this requires the immediate abortion of the procedure, the removal of patient restraints, the repositioning of fluoroscopy equipment, and, finally, the repositioning of the patient into the supine position, often from the fluoroscopy table onto a transport cart. This is a coordinated effort that may result in a delay in providing an advanced airway if not performed swiftly. For this reason, in patients with particular anaesthesiologic risks, the procedure should be performed in the left lateral position or after tracheal intubation. 

### 5.5. ENDOSCOPIC ULTRASONOGRAPHY-GUIDED PANCREATIC DRAINAGE

According to Giovannini, [26] EUS-guided pancreatic pseudocyst drainage should be performed with the patient in the left lateral or prone position. In our experience, it is also possible to use the supine position if it is not necessary to also perform an ERCP during the same procedure.

## 6. Patient Position for Specific Procedures: Lower—GI

A few randomised trials have compared the standard left lateral position with other positions [27,28,29,30,31]. The caecal intubation rate, caecal intubation time, and adenoma detection rate were compared across patients in different decubitus positions, and no significant differences were observed when comparing the left lateral position with the supine and prone positions.

In reality, the patient’s position can be favourably modified during colonoscopy. Position changes during an examination of the colon were first used by radiologists during barium enemas [14], and they are now used in daily practice to facilitate a full examination of the cecum during colonoscopy [32]. For example, with the patient in the left lateral position, if it proves difficult to reach the last few centimetres to the caecal pole, changing the patient’s position to supine can be helpful. 

As reported above, the patient’s position can also influence the distension of the colon during insufflation. Ball et al. [33] studied the effect of patient position changes on colon distension in different segments. According to their results, the hepatic flexure (the right side of the body) is best examined in the left lateral position. The transverse colon (the anterior location in the body) is best examined in the supine position, while the splenic flexure and descending colon (the left side of the body) are best examined in the right lateral position.

As a result, dynamic position changes during colonoscope withdrawal significantly improve polyp and adenoma detection [34,35].

Changes to the patient’s position when performing a difficult polypectomy can make the polypectomy an easier and safer procedure. In such a case, the added time needed to perform this move is compensated by a quicker polypectomy [14].

## 7. Discussion

From the results of this study, it appears that there are few publications regarding the position of the patient during operative endoscopy. The most debated topics concern the position of the patient during ERCP, in particular comparing the prone position with the left lateral position and the variation in the patient’s position during colonoscopy, in order to increase the detection of polyps. In reality, although it is little considered in the literature, the aspect of a patient’s position can be relevant for numerous operative procedures.

In fact, we have seen that the position of the patient can expose the lesion to be treated differently, particularly when it is covered by a collection of liquids, such as in bleeding. In this case, the effect of gravity on intraluminal fluids must be considered. Sometimes, simply changing the patient’s position is enough to make a procedure easier and safer.

In reality, the effect of gravity exerts itself on many elements that can be important during an operative endoscopy procedure. One of these is the terminal of the instrument; in fact, we have seen that when carrying out the posterior myotomy during POEM, if the patient is in a supine decubitus, the shaft tends to lie in a neutral position with less tension on the mucosal opening. Moreover, in advanced sigmoid achalasia, in the left lateral position, the severity of angulations is greater because of the lateral exertion of gravity. This can increase the technical difficulty of submucosal tunnelling and subsequent myotomy [20].

Another element on which the effect of gravity can be exerted is the neoplasm undergoing submucosal dissection. It is known that a limitation of endoscopic submucosal dissection is represented by the lack of a traction system, as normally occurs in surgery. The endoscopist must, therefore, adopt various tricks to facilitate the traction of the piece during the procedure in order to expose the submucosal and muscular layers beneath the lesion. We have seen that some techniques exploit this effect by floating the lesion, as in the underwater ESD technique [10,11].

Finally, some operative endoscopy procedures, exerting their action in depth, can damage organs adjacent to the digestive tract.

Patient position may also play a role in this risk, as in reported cases of accidental gallbladder capture in the suture during the endoscopic sleeve gastroplasty procedure. We have, in fact, seen that, according to some authors, this risk is greater in the case of a left lateral position rather than in a supine position [13].

In the present study, actual NOTES procedures were not included. This is because, in the case of NOTES procedures, the issue can become much more complex. In fact, there are also other elements to consider, such as the orientation outside the gastrointestinal lumen and the exposure of the organs to be treated. It would certainly be interesting to explore these topics in further future studies.

Finally, we have seen that the positioning of the patient during operative endoscopy must also take into consideration any limitations of the patient themself, whether these are anatomical limitations, functional limitations, or recent surgical outcomes. Furthermore, in more fragile patients, it is also necessary to take into account the anaesthesiological risk. We have seen, in fact, that the patient’s position can affect their respiratory function, and the prone position can make it more difficult and time-consuming to intubate the patient if this is deemed necessary.

## 8. Conclusions

The growing operative role of endoscopy has led endoscopists to recognise the importance of the positioning of the patient during procedures. The endoscope’s tip position with respect to the lesion, fluid pooling, organ distension, the risk of damage to adjacent organs, tumour traction during dissection, and the radiological anatomical view are just some of the phenomena that can be conditioned by the patient’s position.

These considerations also have to be taken into account in the organisation of future endoscopy rooms. The presence of mobile operating beds and multiple monitors connected to the endoscopic column can be helpful for operative endoscopists.

## Data Availability

Not applicable.
